# The Use of 7,7′,8,8′-Tetracyanoquinodimethane for the Spectrophotometric Determination of Some Primary Amines Application to Real Water Samples

**DOI:** 10.1155/2013/803767

**Published:** 2013-10-07

**Authors:** Theia'a N. Al-Sabha, Najwa M. Al-Karemy

**Affiliations:** Chemistry Department, College of Education, Mosul University, Mosul 00964, Iraq

## Abstract

A sensitive, simple, and accurate spectrophotometric method was developed for the quantitative determination of some primary aliphatic and aromatic amines, that is, ethylamine, 1,2-diaminopropane, aniline, p-aminophenol, and benzidine. The method is based on the interaction of these amines in aqueous medium with 7,7′,8,8′-tetracyanoquinodimethane (TCNQ) reagent in the presence of a buffer solution and surfactant (in the case of aromatic amines) to form charge-transfer complexes measurable at maximum wavelengths ranging between 323 and 511 nm. Beer's law is obeyed over the concentration ranges of 0.025 and 3.0 **μ**g/mL and the molar absorptivity is ranged between 8.977 × 10^3^ and 5.8034 × 10^4^  L**·**mol^−1^ **·**cm^−1^ for these amines. The method was applied for the determination of benzidine in the river, sea, and tap waters. The TCNQ complexes with the previously mentioned amines were formed in the ratio of 1 : 1 amine : TCNQ, and their stability constants ranged between 8.78 × 10^4^ and 1.844 × 10^5^ L**·**mol^−1^.

## 1. Introduction

A great number of aromatic amines are of considerable importance in industrial, toxicological, and pharmaceutical aspects [1]. Short-chain aliphatic amines are presented widely in the aquatic environment due to their widespread use in several industrial, chemical, and manufacturing applications [2, 3]. Also these amines are common components of biological systems as degradation products of organic materials such as amino acids and proteins. In addition to hygienic problems due to stinging smell, these compounds may be hazardous to human health as they are sensitizers and irritants to skin, eyes, mucus membranes, and respiratory tract. Also they can react with certain nitrogen-containing compounds to form nitrosamines, which are potentially carcinogenic substances [4].

The charge-transfer (CT) reactions had been widely studied spectrophotometrically in the determination of aliphatic and aromatic amines that are easy to be determined based on CT complex formation with some electron *π*-acceptors such as chloranil [5–7], 2,3-dichloro-5,6-dicyano-1,4-benzoquinone (DDQ) [8], fluoranil [9], dinitrobenzene [10], 1-fluro-2,4-dinitrobenzene [11], and 1-chloro-2,4-dinitrobenzene [12]. Most of the previous methods suffer from the lack of sensitivity and selectivity and the estimation of these compounds is applied in organic medium. 7,7,8,8-Tetracyanoquinodimethane (TCNQ) is strong electron acceptor and applied in the determination of several electron donor drugs containing primary, secondary, or tertiary amino group and the review of the literature in the last decade had been mainly concentrated on the CT-complexes spectral studies [13–18].

The present research aims chiefly to develop a sensitive, selective, simple, and quick spectrophotometric method for the determination of samples of primary aliphatic and aromatic amines, namely, ethylamine, 1,2-diaminopropane, aniline, p-aminophenol, and benzidine, in aqueous solution with TCNQ reagent by measuring the absorbance at *λ*_max⁡_ of new charge-transfer absorption band and without any derivatization or catalysis. In addition to applying the method for determination of benzidine in various water samples.

## 2. Experimental

### 2.1. Apparatus

All absorption measurements were made on a Shimadzu UV-210A double-beam spectrophotometer supplied with a digital printer DP80Z and matched 1 cm optical silica cells.

### 2.2. Reagents

All reagents used were of analytical grade and obtained from Fluka and BDH companies.

1. TCNQ solution (1 × 10^−3^ M) is prepared freshly by dissolving 0.0102 g of 7,7′,8,8′-tetracyanoquinodimethane in absolute acetone or acetonitrile solvent, for determination of aromatic or aliphatic amines, respectively, and diluted to the mark in 50 mL volumetric flask with the same solvent.
2. Standard solutions of primary aliphatic and aromatic amines (100 *μ*g/mL) are prepared individually by dissolving 0.01 g of pure amine(ethylamine, 1,2-diaminopropane, aniline, m-aminophenol, and benzidine) in 5 mL ethanol and diluted to the mark with distilled water in 100 mL volumetric flask. These solutions were further diluted with water to required concentrations for working solutions.
3. Phosphate buffer solutions of pH values 6.02, 9.86, and 10.65 were prepared by mixing various volumes of 0.2 M potassium dihydrogen orthophosphate solution with 0.2 M potassium hydroxide solution and adjusted with pH meter type Philips (PW9420).
4. Cetylpyridinium chloride (CPC) and cityltrimethylammonium bromide (CTAB) surfactants were prepared in 0.1% concentration by dissolving 0.1 g in warm distilled water and the volume was completed to 100 mL in calibrated flask.

## 3. General Procedures

### 3.1. Determination of Primary Aliphatic Amines

Aliquots of standard primary amine solutions of ethylamine and 1,2-diaminopropane were transferred separately into a series of 10 mL calibrated flasks. To each of these were added 0.5 and 1.0 mL of 1 × 10^−3^ M TCNQ and the solutions were heated at 40 and 50°C for 20 and 30 min for ethylamine and 1,2-diaminopropane, respectively; then the solutions were cooled to room temperature and diluted to the mark with distilled water. The absorbances of the complexes were measured at 464 and 470 nm for the previous amines, respectively, against corresponding reagent blank.

### 3.2. Determination of Primary Aromatic Amines

 Aliquots of standard primary amine solutions of aniline, p-aminophenol, and benzidine were transferred separately into a series of 10 mL calibrated flasks. To each of these were added the optimum amounts of TCNQ, phosphate buffer solution, and CPC (or CTAB in the case of benzidine) according to the order of addition as listed in Table 3. The solutions were diluted to the mark with distilled water and the absorbances of the complexes were measured at room temperature immediately (in the case of p-aminophenol the solutions were heated at 40°C for 5 min and cooled to room temperature then diluted to the mark with distilled water) at 323, 511, and 500 nm for aniline, p-aminophenol, and benzidine, respectively.

## 4. Results and Discussion

### 4.1. Absorption Spectrum of the CT Complexes

**Figure 1:**
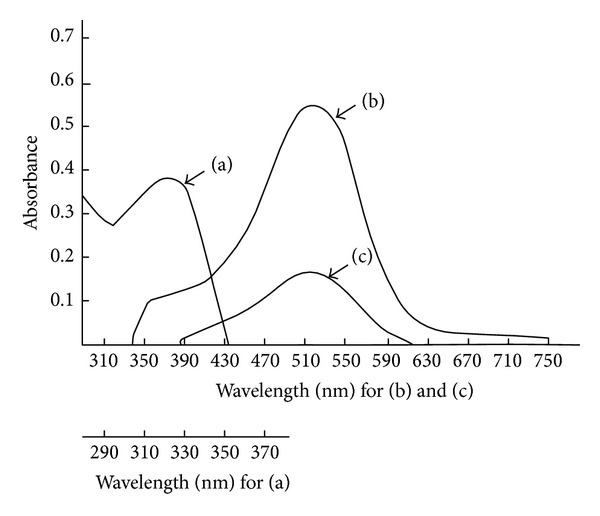
Absorption spectra for the complexes of 0.5 mL of 1 × 10^−3^ M TCNQ with (a) 2 *μ*g/mL aniline, (b) 2 *μ*g/mL p-aminophenol, and (c) 0.75 *μ*g/mL benzidine versus blank reagent.

**Figure 2:**
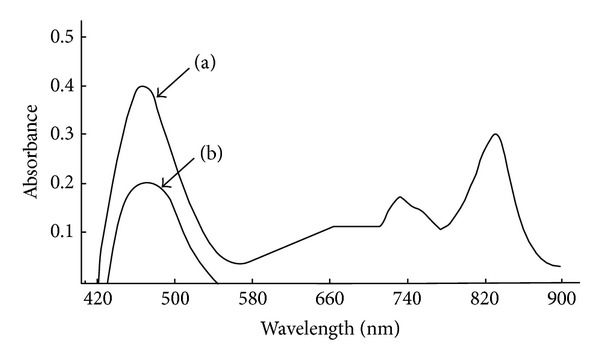
Absorption spectra for the complexes of 0.5 mL of 1 × 10^−3^ M TCNQ and (a) 1 *μ*g/mL ethylamine and (b) 2 *μ*g/mL 1,2-diaminopropane versus blank.

Primary aromatic and aliphatic amines react with TCNQ in the presence of phosphate buffer solution (in the case of aromatic amines) to give a red coloured complexes with maximum absorption spectra at 511, 500, 464, and 470 nm for p-aminophenol, benzidine, ethylamine, and 1,2-diaminopropane, respectively, whereas aniline gave a yellow coloured complex at 323 nm (Figures 1 and 2), and their reagent blanks gave maximum absorption at 320 nm for p-aminophenol and benzidine, whereas they gave it at 395 nm for aniline and 832 nm for ethylamine and 1,2-diaminopropane under their optimum conditions. 

### 4.2. Effect of Solvent

Different solvents such as acetonitrile, acetone, ethanol, methanol, dioxane, and water were tested as reaction media for interaction between amines and TCNQ *π*-acceptor. It was found that TCNQ reagent reacted with primary, secondary, and tertiary aliphatic and aromatic amines in the medium of previous solvents and produced different colours, but using water as a solvent for amines and acetonitrile or acetone for TCNQ and dilution with water, selective reactions between TCNQ and primary aliphatic and aromatic amines occurred and forming coloured *n*-*π* charge-transfer complexes with maximum absorption at wavelengths ranged between 323 and 511 nm for given amines, whereas other amines were either unreacted or gave low absorption response. Therefore, this system of solvents is recommended in our method.

### 4.3. Effect of pH and Buffer Solutions

The effect of pH on the absorption of the complexes produced by the reaction of TCNQ with primary amines was studied using different pHs ranging from 2 to 12. It was found that these complexes are formed in the final pH of 9.86, 6.02, and 10.65 for p-aminophenol, aniline, and benzidine, respectively, by addition of NaOH solution and decreases in absorbances were found through addition of HCl, which may be attributed to the liberation of hydrogen cyanide, whereas it was found that the absorbances of aliphatic amines were decreased through addition of NaOH or HCl and the final pHs of their reaction solutions were 7.20 and 6.62 for ethylamine and 1,2-diaminopropane, respectively. Different buffers of the same pHvalues, as mentioned before, were prepared by using carbonate, borate, phosphate, ammonia, and citrate buffers to investigate the sensitivity of the amine-TCNQ complexes. It was found that phosphate buffer solution (KH_2_PO_4_ + KOH) increased the sensitivity for the aromatic amine-TCNQ complexes (Table 1) and caused a bathochromic shift for p-aminophenol (460→475 nm), whereas the absorbances of aliphatic amine-TCNQ complexes were decreased. However, the amounts of phosphate buffer solution at fixed pH values as cited before for aromatic amines were studied and found to be 0.5, 1.0, and 0.8 mL which are the optimum amounts for aniline, p-aminophenol, and benzidine, respectively, and which are recommended in the subsequent experiments.

### 4.4. Effect of Reaction Time and Temperature

The reaction time was determined by following the color development at room temperature (28°C) and in thermostatically controlled water bath at different temperatures up to 50 ± 1°C. The absorbance was measured at 5- and 10-minute intervals against reagent blank treated similarly. It was observed that the absorbance reached maximum after addition of the reagent solution immediately at room temperature for aniline and benzidine, after 5 min at 40°C for p-aminophenol, and after 20 and 30 min at 40 and 50°C for ethylamine and 1,2-diaminopropane, respectively, and the stability of their absorbances was achieved after cooling to the room temperature (Table 3). These temperatures and reaction times were chosen for colour development.

### 4.5. Effect of TCNQ Concentration

The effect of changing the TCNQ concentration (0.2–2.0 mL of 1 × 10^−3^ M) on the absorbance of solution containing a fixed amount of each primary amine (1.0 *μ*g mL^−1^) was studied. It is evident that the absorbance increases with increasing TCNQ concentration and reached maximum on using 0.5 mL of 1 × 10^−3^ M TCNQ for aniline, p-aminophenol, benzidine, and 1,2-diaminopropane, and 1.0 mL for ethylamine (Table 3). Therefore, these concentrations were used in all subsequent work.

### 4.6. Effect of Surfactant

Effect of various surfactants including CTAB, CPC, tween-80, and triton x-100 of 0.1% concentration was tested. It was found that the cationic surfactants CPC and CATB caused a bathochromic shift with increasing in the sensitivity for aromatic amines (Table 2), while it decreased the sensitivity of aliphatic amines. However, 0.5–3.0 mL of 0.1% CPC were tested on the absorption of 2.5 and 1.5 *μ*g mL^−1^ aniline and p-aminophenol, respectively, and 0.2–2.0 mL of 0.1% CTAB on the absorption of 1.0 *μ*g mL^−1^ benzidine, in the presence of phosphate buffer solution. It was found that 0.5 mL of CPC is the optimum amount for aniline and p-aminophenol and 1 mL of CTAB for benzidine which is recommended in subsequent experiments.

### 4.7. Effect of Order of Addition

In order to obtain the high colour intensity, the order of addition of reagents for aromatic amines should be followed as given in the Table 3; otherwise a loss in colour intensity was observed.

However, the optimum reaction conditions for developing the colour intensity of primary amine-TCNQ complexes are summarized in Table 3.

## 5. Analytical Parameters

Under the experimental conditions described in Table 3, standard calibration curves of CT complexes for aliphatic and aromatic amines with TCNQ were constructed by plotting absorbance versus concentration. The correlation coefficients ranged from 0.9961 to 0.9993, indicating good linearity. Beer's law is obeyed in the ranges cited in Table 4, and the molar absorptivity values indicate the high sensitivity of the method.

## 6. Precision and Accuracy

Six replicate measurements are performed at three different concentrations of each amine. The relative standard deviation and recovery % results indicated the high precision and accuracy of the proposed method (Table 5).

## 7. Interferences

The interference from various organic nitrogen compounds including secondary, tertiary amines and amides in addition to sodium chloride, n-hexane, and glucose on the determination of 1 *μ*g/mL of p-aminophenol (as an example for primary aromatic amines) and 0.5 *μ*g/mL of ethylamine (as an example for primary aliphatic amines) was examined. It was found that these compounds did not affect the recovery % in the range from 5 *μ*g/mL of triethylamine to 2500 *μ*g/mL of acrylamide for ethylamine and from 5 *μ*g/mL of diethylamine to 100 *μ*g/mL of acetanilide for p-aminophenol. The results are summarized in Table 6.

## 8. Application to Real Water Samples

The described method was applied to the analysis of benzidine in tap, river, and sea waters. Three different concentrations 2.5, 5.0, and 10 *μ*g/mL of benzidine were added to the various filtered volumes of tap, Dijlla river, and synthetic sea waters [20] in final volume of 10 mL and treated as described in the previous general procedure. The results in Table 7 indicated that benzidine could be determined in the presence of small volumes of tap and river waters, but an interference was observed in the presence of small volumes of sea water; this may be attributed to the formation of metal-ligand complexes.

## 9. Stoichiometry

**Figure 3:**
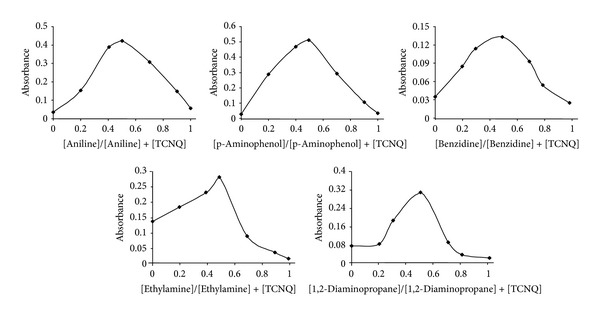
Continuous variation plots of TCNQ complexes with primary aliphatic and aromatic amines.

The stoichiometry of the reaction of primary amines with TCNQ was studied by Job's method [21], using solutions of equimolar (1 × 10^−3^ M) of each primary aliphatic and aromatic amines and TCNQ reagent (1 × 10^−4^ M in the case of benzidine). The results obtained in Figure 3 show that 1 : 1 amine to reagent was formed. This indicates that only one amino group is responsible for the formation of the products.

## 10. Stability Constant of Amine-TCNQ Complexes

The apparent stability constant was estimated by comparing the absorbance of a solution containing stoichiometric amounts of the primary amine and TCNQ (As) to one containing an excessive (optimum) amount of TCNQ reagent (Am). The average conditional stability constants of the complexes are calculated by the following equation:

(1) $$\begin{array}{c} \text{Kc}=1-\frac{\alpha }{\alpha^{2}C},\\ \alpha =\text{Am}-\frac{\text{As}}{\text{Am}}, \end{array}$$

where Kc is the association constant (L·mol^−1^), *α* the dissociation degree, and *C* the concentration of the complex which is equal to the concentration of primary amine. The results shown in Table 8 indicate that the complexes are relatively stable.

## 11. Reaction Mechanism 

**Scheme 1:**
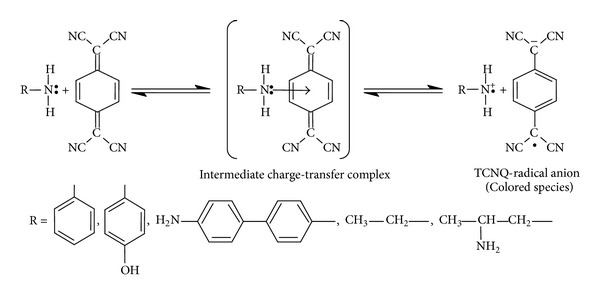
Probable mechanism for the reaction of TCNQ with primary amines.

The nature of the reaction between primary amines in aqueous solution and TCNQ reagent is not clearly understood. Most of the spectrophotometric methods with TCNQ [22–26] are based on the charge-transfer interaction of radical anion TCNQ^.−^ with the radical cation donors (D^.+^) formed in acetonitrile medium leading to enhancement of the absorption bands of TCNQ reagent in acetonitrile solvent at 840, 825, 762, and 742 nm. However, in the present work it was observed that the complexes are formed in aqueous medium in the ratio of 1 : 1 amine : TCNQ with the appearance of a new absorption bands at 464, 470, 323, 511, and 500 nm for ethylamine, 1,2-diaminopropane, aniline, p-aminophenol, and benzidine, respectively, which is not shown by either of the components present in solution which may be attributed to the complete transfer of the unshared pair of electrons on the nitrogen atom to TCNQ reagent and assigned as a charge-transfer complex absorption bands. On this basis, a tentative reaction mechanism has been proposed and given in Scheme 1.

## 12. Comparison with Other Reported Spectrophotometric Methods

A comparison of some parameters, linearity range, and sensitivity of the current method with those of some other reported spectrophotometric methods, using different *π*-acceptors, was described (Table 9). It is quite clear that current method is more sensitive than the reported methods and all the charge-transfer complexes of TCNQ reagent having maximum absorption at visible regions except of TCNQ-aniline complex appears at UV region.

## 13. Conclusion

The proposed method is simple, rapid, sensitive, and economical compared to already reported methods and does not require any pretreatment of the primary amines or extraction procedure and has a good accuracy and precision. On the other hand, in terms of simplicity and expense, the method could be considered superior in comparison with the previously reported methods, especially with those based on nonaqueous medium.

## Figures and Tables

**Table 1:** Effect of buffer solutions on the absorbance of aromatic amine-TCNQ complexes.

Type of buffer solution	Absorbance of TCNQ complex with		
	p-Aminophenol* (5 *μ*g/mL)	Benzidine (1.5 *μ*g/mL)	Aniline (2.5 *μ*g/mL)
Without	0.366	0.254	0.155
Na_2_CO_3_ + NaHCO_3_	0.590	0.368	—
H_3_BO_3_ + NaOH	0.612	0.363	—
Na_2_B_4_O_7_ + NaOH	0.713	0.328	—
KH_2_PO_4_ + KOH	0.696 (0.716**)	0.388	0.220
NH_4_Cl + NH_4_OH	0.695	—	—
Citric acid + sodium citrate	—	—	0.185
Na_2_B_4_O_7_ + HCl	—	—	0.200
NaCl + Na_2_B_4_O_7_ + H_3_BO_3_	—	—	0.216

*Absorbance at 460 nm.

**Absorbance at 475 nm.

**Table 2:** Effect of surfactants on the absorption of aromatic amine-TCNQ complexes.

Surfactant (0.1%)	Absorbance of TCNQ complex with					
	Aniline (2.5 *μ*g/mL) at		p-Aminophenol (1.5 *μ*g/mL) at		Benzidine (1 *μ*g/mL) at	
	313 nm	323 nm	475 nm	511 nm	480 nm	500 nm
CPC	0.230	0.313	0.242	0.400	0.236	0.246
CTAB	0.172	—	0.228	—	0.254	0.268

**Table 3:** Optimum conditions for the determination of primary amines with TCNQ reagent.

Compound	*λ*_max⁡_ (nm)	Temp. (°C)	Development time (min)	Buffer amount (mL)	TCNQ 1 × 10^−3^ M (mL)	Order of addition	Surfactant 0.1% (mL)	Final pH
Aniline	323	RT^a^	Immediately	0.5	0.5	III^b^	CPC, 0.5	6.02
p-Amino-phenol	511	40	5	1.0	0.5	III	CPC, 0.5	9.86
Benzidine	500	RT	Immediately	0.8	0.5	IV^c^	CTAB, 1.0	10.65
Ethylamine	464	40	20	—	1.0	—	—	7.20
1,2-Diaminopropane	470	50	30	—	0.5	—	—	6.62

^a^Room temperature = 25°C.

^b^Amine + (KH_2_PO_4_ + KOH) + TCNQ + CPC.

^c^TCNQ + (KH_2_PO_4_ + KOH) + CPC + amine.

**Table 4:** Summary of optical characteristics and statistics for the proposed method.

Parameters	Complex of TCNQ with				
	Ethylamine	1,2-Diaminopropane	Aniline	p-Aminophenol	Benzidine
*λ*_max⁡_ (nm)	464	470	323	511	500
Linearity range (*μ*g/mL)	0.025–1.25	0.25–3.0	0.5–2.5	0.05–2.5	0.05–1.25
Limit of detection (ng/mL)	6.62	22.00	14.44	13.47	11.88
Slope	0.4051	0.1213	0.1857	0.1990	0.2256
Intercept	0.0078	−0.0363	−0.0371	0.0888	0.0227
Correlation coefficients	0.9988	0.9986	0.9993	0.9961	0.9922
Molar absorptivity (L·mol^−1^·cm^−1^×10^4^)	1.8231	8.977	1.7273	2.1699	5.8034

**Table 5:** Precision and accuracy data for primary amines determination obtained by the proposed method.

Compounds	Amount added (*μ*g/mL)	Recovery* (%)	Average recovery (%)	RSD*
	0.5	104.0		2.903
Aniline	1.5	101.3	101.7	0.693
	2.5	100.0		3.689

	0.5	100.0		2.446
p-Aminophenol	1.0	97.5	99.5	0.539
	2.0	101		0.776

	0.15	100.0		1.509
Benzidine	0.5	100.0	99.1	4.383
	1.0	97.5		0.322

	0.125	100.0		5.625
Ethylamine	0.5	105.0	102.5	1.079
	1.0	102.5		0.619

	0.5	100.0		6.153
1,2-Diaminopropane	1.5	100.0	99.3	1.985
	2.5	98.0		0.308

*Average of six determinations.

**Table 6:** Effect of foreign compound on the recoveries of ethylamine and p-aminophenol.

Foreign compound	Ethylamine (0.5 *μ*g/mL)		p-Aminophenol (1 *μ*g/mL)	
	Amount added (*μ*g/mL)	Recovery (%)	Amount added (*μ*g/mL)	Recovery (%)
Diethylamine	20	98.6	5	98.0
Diphenylamine	55	97.2	20	103.0
Triethylamine	5	96.8	10	107.0
Dimethylaniline	250	99.0	10	99.1
Acetanilide	1000	100.7	100	99.6
Acrylamide	2500	100.7	25	101.8
n-Hexane	1000	102.0	100	101.4
NaCl	500	96.0	5	104.2
Glucose	2500	95.6	25	100.9

**Table 7:** Determination of benzidine in different waters.

Water	Benzidine added (*μ*g/mL)	Recovery % of benzidine found per mL of water					
		0.05	0.1	0.3	0.5	1.0	1.5
Tap water	2.5	98.3	98.3	100.0	132.2	153.2	177.4
	5.0	92.0	114.4	113.6	117.6	136.0	142.6
	10.0	101.2	101.2	102.1	102.5	104.5	114.9

River water	2.5	100	103.3	105.0	108.3	158.3	170.0
	5.0	96.7	97.4	97.4	98.07	112.1	117.9
	10.0	92.0	94.9	113.0	119.3	121.4	123.1

Sea water	2.5	128.7	145.4	151.5	215.1	360.6	403.0
	5.0	106.5	129.5	209.8	255.7	362.2	394.2
	10.0	100.0	125.7	163.0	167.8	213.7	223.6

**Table 8:** Association constants of the TCNQ-amine complexes.

Primary amine	Volume (mL)	Conc. (M)	Absorbance		*α*	Kc average (L·mol^−1^)
			As	Am		
	0.1		0.094	0.165	0.430	
Aniline	0.3	1 × 10^−3^	0.213	0.345	0.382	2.741 × 10^5^
	0.4		0.373	0.485	0.229	

	0.05		0.070	0.168	0.583	
p-Aminophenol	0.2	1 × 10^−3^	0.335	0.466	0.281	1.701 × 10^6^
	0.4		0.555	0.599	0.073	

	0.03		0.029	0.043	0.326	
Benzidine	0.1	1 × 10^−4^	0.037	0.073	0.493	1.844 × 10^6^
	0.2		0.057	0.105	0.457	

	0.05		0.037	0.062	0.403	
Ethylamine	0.2	1 × 10^−3^	0.043	0.168	0.744	8.78 × 10^4^
	0.4		0.163	0.240	0.320	

	0.1		0.012	0.097	0.876	
1,2-Diaminopropane	0.3	1 × 10^−3^	0.154	0.195	0.210	8.208 × 10^5^
	0.4		0.188	0.212	0.113	

**Table 9:** Comparison of current method with other reported methods using *π*-acceptors.

*π*-Acceptors	Amine	*λ*_max⁡_ (nm)	Temp. (°C)	Developing time (min)	Linearity range (*μ*g·ml^−1^)	Molar absorptivity (L·mol^−1^·cm^−1^)	Ref.
DDQ*	Aniline	345	50	30	0.1–4.0	1.36 × 10^3^	[8]
	p-Aminophenol	355	40	40	0.2–6.4	3.36 × 10^3^	

p-Chloranil	Aniline	355	55	30	0.2–3.2	1.16 × 10^4^	[7]
	p-Aminophenol	345	20	60	2–32	3.80 × 10^3^	

TCNE**	Ethylamine	325	40	20	0.2–7.2	2.83 × 10^3^	[19]
	Aniline	344	40	20	0.2–7.2	1.23 × 10^4^	
	p-Aminophenol	350	20	5	1–24	4.57 × 10^3^	

TCNQ	Aniline	323	RT	Immediately	0.5–2.5	1.72 × 10^4^	Present method
	p-Amino phenol	511	40	5	0.05–2.5	2.16 × 10^4^	
	Benzidine	500	RT	Immediately	0.05–1.25	5.80 × 10^4^	
	Ethylamine	464	40	20	0.025–1.25	1.82 × 10^4^	
	1,2-Diaminopropane	470	50	30	0.25–3.0	8.97 × 10^4^	

*2,3-Dichloro-5,6-dicyano-1,4-benzoquinone.

**Tetracyanoethylene.
